# Targets of Immune Escape Mechanisms in Cancer: Basis for Development and Evolution of Cancer Immune Checkpoint Inhibitors

**DOI:** 10.3390/biology12020218

**Published:** 2023-01-30

**Authors:** Shovan Dutta, Anirban Ganguly, Kaushiki Chatterjee, Sheila Spada, Sumit Mukherjee

**Affiliations:** 1 The Center for Immunotherapy & Precision Immuno-Oncology (CITI), Lerner Research Institute, Cleveland Clinic, Cleveland, OH 44195, USA; 2 Department of Biochemistry, All India Institute of Medical Sciences, Deoghar 814152, India; 3 Department of Medicine, Weill Cornell Medicine, New York, NY 10021, USA; 4 Department of Radiation Oncology, Weill Cornell Medicine, New York, NY 10065, USA; 5 Department of Cardiothoracic and Vascular Surgery, Albert Einstein College of Medicine, Bronx, NY 10461, USA

**Keywords:** cancer treatment, immune response, cancer therapeutic strategy, tumor immune escape, immune-oncology, tumor immune microenvironment, immune checkpoint inhibitors, mRNA cancer immunotherapy, CRISPR-Cas9 cancer immunotherapy

## Abstract

**Simple Summary:**

The tumor immune escape mechanisms are key factors in cancer progression and metastasis. They are an undeniable hurdle for successful cancer treatment in patients. It has been widely recognized that cancer cells can escape immune surveillance and antitumor immunity. Despite host immunity, tumor cells can escape antitumor immune cell responses by several different mechanisms, such as the loss of the antigen presentation capacity by some immune cells, which promotes tumor progression and resistance to immunotherapy. A few monotherapies or combinational therapies have been approved for use in cancer treatment, but the majority of patients are not responsive to currently used immunotherapies, thus presenting a need to discover new targets to achieve efficacious immune responses to benefit cancer patients. This review focuses on some of the most important classical immune checkpoint targets and also sheds light on some of the recently discovered, promising immunotherapeutic targets and strategies.

**Abstract:**

Immune checkpoint blockade (ICB) has emerged as a novel therapeutic tool for cancer therapy in the last decade. Unfortunately, a small number of patients benefit from approved immune checkpoint inhibitors (ICIs). Therefore, multiple studies are being conducted to find new ICIs and combination strategies to improve the current ICIs. In this review, we discuss some approved immune checkpoints, such as PD-L1, PD-1, and CTLA-4, and also highlight newer emerging ICIs. For instance, HLA-E, overexpressed by tumor cells, represents an immune-suppressive feature by binding CD94/NKG2A, on NK and T cells. NKG2A blockade recruits CD8+ T cells and activates NK cells to decrease the tumor burden. NKG2D acts as an NK cell activating receptor that can also be a potential ICI. The adenosine A2A and A2B receptors, CD47-SIRPα, TIM-3, LAG-3, TIGIT, and VISTA are targets that also contribute to cancer immunoresistance and have been considered for clinical trials. Their antitumor immunosuppressive functions can be used to develop blocking antibodies. PARPs, mARTs, and B7-H3 are also other potential targets for immunosuppression. Additionally, miRNA, mRNA, and CRISPR-Cas9-mediated immunotherapeutic approaches are being investigated with great interest. Pre-clinical and clinical studies project these targets as potential immunotherapeutic candidates in different cancer types for their robust antitumor modulation.

## 1. Introduction

The progression of tumor growth and metastasis is dependent upon a complex interplay between the host immune system and counter-regulatory immune escape pathways implemented by the tumor itself. The host immune system possesses a strong surveillance system that recognizes and eliminates malignant cells and thus forms the basis of cancer immunotherapy, which focuses on boosting such antitumor immune responses to halt cancer progression [1,2,3]. However, the tumor cells gradually develop mechanisms to escape this immune surveillance, which is termed “cancer immunoediting”, to prevent elimination from immune cells with antitumor properties [4]. In general, tumor cells undergo many genetic and epigenetic changes, resulting in the formation of neoantigens, which in turn trigger T cells [5]. This generates a population of cytotoxic T lymphocytes (CTLs), which effectively coordinates to recognize and kill cancer cells [6]. The immune checkpoint molecules are targeted by cancer cells to inhibit T cell activation and upregulate negative signals through cell surface molecules to facilitate cancer progression and metastasis [7]. Some tumor cells may also activate immunosuppressive leukocytes to create a tumor microenvironment that poorly responds to antitumor immune molecules [8]. Several clinical trials and studies are now trying to utilize checkpoint pathways inhibiting antibodies to counteract the immune escape phenomenon and subsequently treat cancers. Research on negative immunomodulation won James P Allison and Tasuku Honjo the Nobel Prize in Physiology/Medicine in 2018. Their research showed that programmed cell death protein 1 (PD-1), along with programmed death ligand 1 (i.e., PD-L1) and cytotoxic T-lymphocyte-associated protein 4 (CTLA-4), blocked immune checkpoints, resulting in the reactivation of T cells and subsequent effective malignant cell elimination [9]. T cell activity at an early stage is principally regulated by CTLA-4, whereas PD-1 mainly acts at a later stage in modulating the tumor microenvironment by restricting the action of T cells [10]. Hence, in developing an effective immunotherapy, PD-1 and its ligands have emerged as very important new targets. A few monotherapies, such as PD-1, or combinational therapies have been approved for use in cancer treatment [11,12,13]. Though immune checkpoint blockade (ICB) has been used as a strategy to boost antitumor immunity and decrease the tumor burden, its successes are unfortunately still restricted to a small number of cancer patients [14]. Relevant efforts are ongoing to overcome this and discover other immune checkpoints to improve the patient response to immunotherapy. To achieve this aim, novel immune checkpoints have been identified and are emerging as successful and promising targets in cancer immunotherapy [11,12,13]. Multiple studies have been conducted to find strategies for improving the response to ICB therapy. For example, NKG2A, the newly discovered inhibitory receptor expressed on subsets of cytotoxic lymphocytes, engages with the non-classical molecule HLA-E. Blocking NKG2A helps to recruit CD8+ T cells and activated NK cells in the tumor microenvironment [15,16,17]. Here, we highlight traditional immune checkpoint inhibitors (ICIs) that are already in use in clinical settings and, additionally, newer, and emerging targets that show promising pre-clinical and clinical results.

## 2. Overview of Immune Checkpoint Inhibitors

### 2.1. PD-1/PD-L1 Axis as an Immune Checkpoint Target

#### 2.1.1. PD-1/PD-L1: Structure, Function, and Overview of Pathway

In the tumor microenvironment, the PD-1/PD-L1 pathway is a vital regulator for inducing and maintaining immune tolerance [18]. PD-1 is a type 1 trans-membrane protein, belonging to the extended group of the CD28/CTLA-4 immunoglobulin (Ig) family [19]. The 288-amino-acid-long PD-1 protein possesses an extracellular Ig-V-like N-terminal domain, a hydrophobic transmembrane region, and an intracellular cytoplasmic tail with two potential phosphorylation sites within two tyrosine residues: namely an immune receptor tyrosine-based inhibitory motif (ITIM) and immune receptor tyrosine-based switch motif (ITSM) [20]. Several mutagenetic studies have shown that the inhibitory effect of PD-1 on T cells is mediated by activated ITSM [21]. Being involved in the inhibition of both innate and adaptive immunity, PD-1 is expressed on activated T cells, B cells, natural killer (NK) cells, monocytes, dendritic cells (DCs), and several tumor-infiltrating lymphocytes (TILs) [22]. It is also shown to be expressed in regulatory T cells (Tregs), where it augments their proliferation and inhibits the immune response [23].

There are two ligands for PD-1, namely PD-L1 (also named B7-1; CD 274) and PD-L2 (B7-DC; CD273). Pancreatic islet cells, the vascular endothelium, antigen presenting cells (APC) such as DCs and macrophages, resting T cells, and B cells are shown to express PD-L1. PD-L1 expression is conspicuous in multiple tumors, such as gastric cancers, leukemias, melanomas, non-small cell lung cancer, renal cell carcinoma, and many other cancers [24,25,26]. PD-L2 is usually expressed in APC and is far less expressed in tumor cells compared to PD-L1 [27,28]. PD-L2 binds to PD-1 with three-times more affinity than PD-L1.

Several pro-inflammatory molecules, including interferon-γ (IFN-γ), cytokines such as interleukin-10 (IL-10), interleukin-4 (IL-4), tumor necrosis factor-α (TNF-α), and vascular endothelial growth factor (VEGF), play a significant role in upregulating the expression of PD-L1 [29,30]. Protein kinase D isoform 2 (PKD2) is an important regulator of PD-L1 and this enzyme is induced by IFN-γ, making PKD2 a strong targetable candidate for inhibition to enhance the antitumor immune response [30,31]. The inherent immune response is evident from the regulation of PD-L1 expression by oncogenes and its suppression by the tumor suppressor gene PTEN. Different studies have shown that PTEN deletion in neuroglioma cells increases PD-L1 expression via the activation of the PI3K/AKT downstream mTOR-S6K1 signaling pathway [32]. Alternatively, one of the studies using murine mechanistic models showed that PD-L1 induction in the melanoma tumor microenvironment is mediated by CD8+ T cells and IFN-γ, highlighting that, in some cases, upregulation of PD-L1 is independent of regulation by oncogenes, rather related to CD8+ T cells [24]. Thus, it is evident that PD-L1 functions as a pro-tumorigenic factor via its attachment to different receptors, leading to the activation of signaling pathways related to survival and proliferation [33]. PD-L1 can also exert non-immune proliferative effects on certain types of cancer, such as that in renal cell carcinoma [34]. Epithelial-to-mesenchymal transition (EMT) and stem-cell-like phenotypes are shown to be induced by PD-L1, promoting renal cancer progression [35].

**Figure 1:**
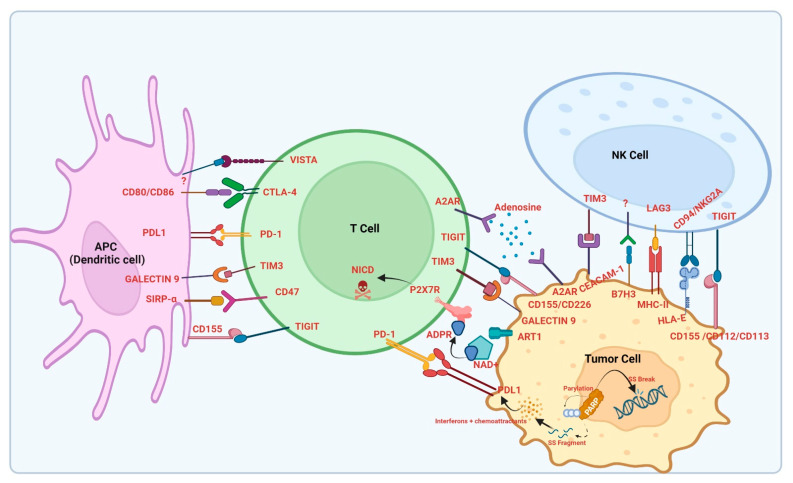
Key immune checkpoint targets for cancer immunotherapy mentioned in this review. Immune checkpoint inhibitors (ICIs) and their respective ligands are reported in the context of the tumor immune microenvironment (TME). Various immune checkpoint target-mediated interactions between immune cells such as dendritic cells (DC) (serving as APCs), T cells, NK cells, and tumor cells are shown here. Mechanisms of action of all the checkpoint proteins mentioned here have been elaborated in their respective sections in this review. ICIs targeting these immune checkpoints are currently used in clinic or under pre-clinical or clinical investigation. The “?” indicates interactions which are unknown/uncertain. This figure has been created with BioRender.com, access date 22 January 2023.

The PD-1/PD-L1 pathway decreases the T cell response by regulating overlapping signals in immune checkpoint gateways. Immune self-tolerance is maintained by the inhibitory immune activity of Tregs that highly express PD-1. In normal conditions, this self-tolerating activity is important to prevent healthy tissue damage during the activation of the immune system by any infection [36]. Cancer cells utilize this machinery to escape the immune system. PD-L1 and PD-L2 expressed by tumor cells bind to the PD-1 receptor on T cells, causing the inhibition of T cell activation, and the subsequent T cell attack is prevented, providing tumor cells with a means to escape the body’s immune surveillance and develop a tumor microenvironment beneficial for its uncontrolled proliferation [37] (Figure 1).

#### 2.1.2. Relationship among Signaling Pathways and PD-1/PD-L1 in Cancer

Different signaling pathways exert their effects on the PD-1/PD-L1 axis, resulting in modulating the progression of tumorigenesis in a variety of cancers. The PI3K/AKT pathway is involved in the regulation of apoptosis and cell proliferation mechanisms [38] and the mammalian target of rapamycin (mTOR) pathway is shown to regulate the immune and adaptive immune systems [39]. In gastrointestinal stromal tumors, it has been shown that the apoptosis of CD8+ T cells can be attenuated by blocking the PD-1/PD-L1 axis, acting through the regulation of the PI3K/AKT/mTOR pathway [40,41].

MAPK is also shown to be associated with PD-L1 expression in lung adenocarcinoma, where inhibition of the MAPK pathway prevented the upregulation of PD-L1 protein and IFN-γ-induced CD274 mRNA [42]. When the MAPK pathway is inhibited, it leads to the markedly reduced expression of PD-L1 in renal cell carcinoma [43]. Recent reports have shown that the JAK-STAT pathway is linked to PD-L1 upregulation, as evidenced by the suppression of PD-L1 upregulation by AG490, a JAK-2 inhibitor molecule [44]. Many studies have shown that abnormal wingless-related integration site (WNT) signals can promote immune escape and lead to resistance developed against different types of immunotherapies [45]. Functional crosstalk between WNT activity and PD-L1 expression is the basis for the use of selective WNT activators or inhibitors to up- or downregulate PD-L1 expression, respectively, in triple-negative breast cancer (TNBC) stem cells, holding the promise of immunotherapy for this cancer [46]. The nuclear factor kappa beta (NF-κβ) is known to modulate IFN-γ-induced PD-L1 expression [47]. In ovarian cancer, chemotherapy is shown to induce PD-L1 upregulation mediated via the NF-κβ pathway [48]. It has been recently reported that Hedgehog signaling helps to induce PD-1/PD-L1 signaling in gastric carcinoma, making the Hedgehog pathway a potential therapeutic target to tackle gastrointestinal cancer [49].

Many other mechanisms regulate PD-1/PD-L1 expression. Several studies have tried to find the association between different miRNAs and PD-1/PD-L1 pathways in the regulation of tumor escape. The regulatory effect of miRNAs takes place by direct binding to the mRNA of PD-L1, as well as indirect regulation of PD-L1 expression. In parallel, recent studies have also shown that a small group of lung cancer patients with low PD-1 expression and high miR-33a levels had a better treatment outcome, indicating the better prognostic value of miR-33a via PD-1 regulation. Hence, this novel mechanism of tumor immune evasion regulated by miR-33a via PD-1/PD-L1 holds great promise as a therapeutic target [50,51].

MiRNAs, such as miRNA28 and miRNA-138, inhibit PD-1 expression in melanoma, glioblastoma, and hepatic cell carcinoma, respectively. Additionally, miRNA15a-bmiRNA16 and miRNA193-3p; miRNA34a-b-c; the cluster of miRNA25, miRNA93, and miRNA106b, 138-5p, 142-5p, and 146-a; miRNA152, miRNA200, and miRNA424 abrogate PD-L1 expression in many different cancer types [52,53,54,55,56,57,58,59,60,61,62].

Furthermore, the regulation of PD-1/PD-L1 expression at the RNA level is also due to the activity of long noncoding RNAs (lncRNAs). For instance, lncRNAs MALAT1, SNHG12, CASC11, PMSB8-AS1, FGD5-AS1, and PCED1B-AS1 increase PD-L1 expression. Interestingly, PCED1B-AS1 transport by exosomes from hepatocellular carcinoma (HCC) cells regulates PD-L1 and PD-L2 in the recipient cells once uptaken [62,63,64,65,66,67]. In addition, the expression of PD-L1 is shown to be induced by the secretion of cytokines by monocytes stimulated by the non-coding small RNA Yh4 in exosomes, acting via the toll-like receptor 7 (TLR7) pathway [68]. Polarization of macrophages to the M2 phenotype can be brought about by tumor-derived exosomes, and this leads to the increased expression of PD-L1 via STAT3 phosphorylation in M2 macrophages, with a further enhancement in immunosuppressive effects [69]. It has also been shown that tumor-derived exosomes having PD-L1 protein exert strong immunosuppressive effects [70]. On the contrary, RNAs NKX2-1-AS1 reduce PD-L1 expression [71]. A specialized class of non-coding RNA molecules with a closed-loop structure, circRNAs are rich in miRNA binding sites and can rescue the target gene from miRNA inhibition by acting as miRNA sponges [72,73,74,75]. One of the initial studies exemplifying the regulation of PD-L1 expression by circRNA was that of Hsa_circ_0020397 (circRNA molecule), exerting an RNA sponge effect and inhibiting the activity of miR-138, leading to an enhancement in PD-L1 expression [76]. Moreover, IFN-γ-dependent PD-L1 expression in xenograft tumors in vivo was induced by anti-lnc RNA urothelial carcinoma-associated 1 (UCA1) targeted therapy [77].

#### 2.1.3. Treatments Targeted at PD-1/PD-L1 Pathway: Role in Cancer Immunotherapy

Researchers have shown recently that cancer immunotherapy targeting the PD-1/PD-L1 pathway has led to an effective and durable antitumor immune response with much lower toxicity in a variety of cancer types [78]. Targeting of the PD-1/PD-L1 signaling pathways is mainly performed to normalize the immune system, rather than a simple enhancement in immune cells in tumors [79]. Anti-PD-1 antibodies have been approved by the FDA since 2014 and are being used in certain cancers. Nivolumab, a human monoclonal anti-PD-1 antibody, has been approved for use in unresectable or metastatic melanoma [80], metastatic NSCLC [81], Hodgkin’s lymphoma [82], and hepatocellular carcinoma [83]. Pembrolizumab, another human monoclonal anti-PD-1 antibody, has been approved for the treatment of metastatic melanoma and sometimes for non-Hodgkin’s lymphoma and head and neck squamous cell carcinoma [84,85,86]. In addition, anti-PD-1 Cemiplimab is a therapeutic agent for advanced cutaneous squamous cell carcinoma [87]. In parallel, several anti-PD-L1 monoclonal antibodies have also been made commercially available for use. Atezolizumab has been used for urothelial carcinoma, renal cancer, bladder transitional cell carcinoma, and breast cancer [88,89,90]. Avelumab, another anti-PD-L1, reactivates T cells and induces antibody-dependent cell-mediated cytotoxicity (ADCC) via its native Fc region. It has been recommended for use in Merkel cell carcinoma [91]. Duravulumab, another monoclonal anti-PD-L1 antibody blocking PD-L1/PD-1 interaction, prevents the immune escape of tumors. It has been used for the treatment of HNSCC [92] (for more key clinical studies, please refer to Table 1).

### 2.2. Cytotoxic T Lymphocyte-Associated Antigen (CTLA-4) as an Immune Checkpoint Target

#### 2.2.1. Structure and Basic Role of CTLA-4 in Immune Checkpoint

CTLA-4 (also known as CD152) is a transmembrane protein that has an extracellular surface receptor and a cytoplasmic domain having two tyrosine motifs mediating signal transduction. The surface receptor closely resembles CD28, which facilitates competitive binding [93]. CTLA-4 is normally expressed in Treg cells and T-anergic cells [93], but Treg cells constitutively express CTLA-4 due to increased levels of FoxP3 [94,95,96]. Regulation of T cell function and prevention of immune cell-mediated damage of normal tissues is mediated by CTLA-4 [93]. Activation of T cell receptor (TCR) leads to the trafficking of CTLA-4 to the cell membrane mediated by T-cell interacting molecules (TRIM), a unique disulfide-linked dimer associated with the TCR-CD3-zeta complex. It further becomes phosphorylated and remains attached to the cell surface [97]. Being a cell surface receptor related to CD28, CTLA-4 binds to ligands CD80 and CD86 on antigen-presenting cells (APCs) [98]. In naïve T cells, CTLA-4 is not detectable but it is highly induced upon T cell activation and as such acts as a primary regulator of T cell amplitude in lymphoid organs during the early priming phase [97,99]. CD28 receptors present on T cells bind to B7 ligands on APCs during the process of T cell activation and hence provide the second activation signal to T cells [100]. Studies have shown that CTLA-4 receptors outcompete CD28 receptors in binding to B7 ligands and hence there is an absence of the second activation signal in the presence of CTLA-4 receptors, leading to anergy in T cells [101,102,103]. Besides having higher affinity towards B7 ligands compared to CD28, CTLA-4 receptors have also been demonstrated to sequester B7 ligands from the surfaces of the APCs, leading to a substantial decrease in ligands on their surfaces. Hence, it is obvious that whether a T cell will be activated or enter anergy depends to a great extent on the relative amount of CD28:B7 binding versus CTLA-4:B7 binding [98]. Multiple interactions with GRB2, PI3K, PKC, PTPN11, filamin A, ZAP70, and PP2A have been reported for CTLA-4, all of which are pivotal for inhibitory response initiation within T cells [14] (Figure 1). Activation of CTLA-4 has been shown to inhibit interleukin-2 (IL-2) production and T cell proliferation and induce cell cycle arrest by mediating crosstalk with other pathways linked to cell proliferation and survival, such as the NF-κβ, PI3K, and MAP kinase pathways [104,105,106,107,108].

#### 2.2.2. Negative Co-Stimulation Mediated by CTLA-4

CTLA-4 not only primarily regulates T cell activity at the sites of T cell priming but also attenuates T cell activation in peripheral tissues. Indeed, CTLA-4 is critical for “tolerance”, a fact substantiated by the experimental finding that the biallelic deletion of the *Ctla4* gene led to massive lymphoproliferative disorder in mice [109,110,111]. Besides cell-intrinsic mechanisms for the regulation of T cell activity, CTLA-4 can also act via cell-extrinsic mechanisms. Studies have demonstrated the existence of these cell-extrinsic mechanisms by showing that lethal lymphoproliferation after the genetic deletion of *Ctla4* can be prevented by CTLA-4-competent T cells [112]. Tregs have been shown to be the main mediators of the cell-extrinsic suppressive function of CTLA-4 [113,114]. Additionally, it has been shown that CTLA-4 can limit the availability of B7 ligands through trans-endocytosis from APCs [115]. Research has shown that in Tregs, genetic loss of CTLA-4 during adulthood confers resistance to experimental autoimmune encephalitis, which suggests that the unrestrained peripheral expansion of Tregs and/or increased activation of Tregs can prevent autoimmunity [116]. More work needs to be done to understand to what extent tumor immunity is regulated by T cell tolerance mediated by cell-extrinsic processes.

#### 2.2.3. Therapeutic Potential of CTLA-4 Blockade Therapy in Cancer

Studies on murine tumor models highlighted the noteworthy anticancer potential of CTLA-4 blockade, which led to the promotion of anti CTLA-4 antibodies [117]. As early as 1996, one study showed that when mice with pre-established tumors were injected with anti-CTLA-4 antibody, tumor growth was reduced significantly [118]. Primarily, the direct blockade of CTLA-4 removes competition for B7-1 and B7-2 costimulatory ligands, allowing unrestrained CD-28-mediated positive co-stimulation [119]. Blocking CTLA-4 affects the immune priming phase by reducing the Treg-mediated suppression of T cell responses and by supporting the activation and proliferation of an increased number of effector T cells irrespective of TCR specificity [101]. Ipilimumab, the first immune checkpoint inhibitor and CTLA-4 blocker to receive FDA approval, has been successfully used for the treatment of metastatic melanomas [120]. Ipilimumab has been approved for use along with PD-L1 inhibitor nivolumab for the treatment of unresectable melanoma, renal cell carcinoma, and other tumors [121]. Tremelimumab is another CTLA-4 blocker acting similarly to ipilimumab and thereby inhibiting CTLA-4-mediated immune cell inactivation by blocking the interaction between CD28 and CTLA-4 [122]. Being considered an orphan drug for the treatment of malignant mesothelioma, tremelimumab has been considered to be used along with several immunomodulatory agents in several other cancers [118]. CTLA-4 blockade has been postulated to modulate the T cell repertoire, as is evident from the remodeling and broadening of the peripheral TCR repertoire with the use of ipilimumab [123,124]. Further studies are required to understand the precise mechanisms of the benefits rendered by CTLA-4 blockade and to make it more suitable as a combination therapy with other immune checkpoint inhibitors (for more key clinical studies, please refer to Table 1).

### 2.3. HLA-E/NKG2A Axis as an Immune Checkpoint Target

#### 2.3.1. Relationship between Tumor Microenvironment and HLA-E

The human leukocyte antigen (HLA) is also known as the human version of the major histocompatibility complex (MHC). MHC is in turn divided into classes I and II. HLA-E is a non-classical MHC class I molecule that plays a critical role in the immune response by both inhibiting and activating the function of NK cells and T cells [125]. Immunosuppression occurs upon its binding to inhibitory NKG2A receptors on both T cells and NK cells. Inhibiting this interaction between HLA-E and NKG2A can serve as a potential immunotherapeutic strategy [125].

Moreover, MHC class I includes three major and three minor genes in the HLA locus. HLA-A, HLA-B, and HLA-C belong to major MHC class I, whereas HLA-E, HLA-F, and HLA-G are minor genes. HLA class I is constitutively expressed on all cell types, including tumor cells. It presents endogenous processed antigens to the immune system, regulating CD8+ T cell and NK cell activation. HLA class II is expressed by APCs and presents exogenous antigens to T helper cells [126]. In cancer, HLAs present tumor antigens, which are recognized by T cells, to facilitate the immune system to recognize tumor cells [127]. Since classical HLA class I molecules are recognized by tumor-specific cytotoxic CD8+ T lymphocytes, the downregulation or loss of HLA class I molecules will enable tumors to escape from T-cell-mediated immune responses [128]. It has been reported that class I molecules can function as tumor suppressor genes in melanoma. Downregulating the class I gene enhanced the carcinogenicity of cells and allowed melanoma cells to enhance their proliferation, migration, and invasion [129,130].

In cancers, classical HLA class I molecules are lost to prevent T-cell-mediated recognition but, interestingly, the expression of HLA-E molecules is enhanced [131,132,133,134]. HLA-E functions as an immunomodulatory molecule by binding to the receptors CD94/NKG2A, -B, and -C on NK and T cells [135,136,137,138]. NKG2A belongs to the NKG2 family protein, a transmembrane C-type lectin-like receptor superfamily, also known as CD159 [139]. NKG2A dimerizes with CD94 to become an inhibitory receptor [140]. NKG2A and its splice variant NKG2B contain ITIMs in the intercellular part of the molecule. The interaction of NKG2A/CD94 with peptide-loaded HLA-E leads to the phosphorylation of ITIM, recruiting phosphatases such as SHP-1 to the signal-transducing synapse, resulting in decreased effector functions [141,142] and in the transmission of an inhibitory signal [143] (Figure 1).

#### 2.3.2. Interaction of HLA-E with Immune Cells

Studies have shown that HLA-E has a role in both tumor escape and tumor immune surveillance. Especially in colorectal and breast carcinomas, a negative prognostic association of HLA-E expression seems to facilitate tumor escape. During this immune escape process, the selective loss of HLA-A, HLA-B, and HLA-C alleviates and stabilizes the expression of HLA-E that engages the inhibitory NKG2A receptor, further promoting immune escape [128,131,132,144].

However, few studies show HLA-E to have a purely negative role in survival. Further studies have also argued that HLA-E is widely expressed in human tumors and, additionally, high HLA-E expression seems to correlate with good prognosis in melanoma and glioblastoma [145,146].

It is now a well-known fact that HLA-E expression has clinical relevance, and it has a high impact on tumor progression, metastasis, and the reduced survival of patients with some tumors, e.g., in laryngeal [147], mammary [131], non-small-cell lung [148], ovarian [149], and colorectal carcinomas [150,151]. In hematopoietic malignancies, the HLA-E surface was characterized by a high percentage of lymphoid tumor cells and this played a protective role regarding NK cell-mediated cytotoxicity [128]. Acute myeloid leukemia (AML) cells treated with IFN-γ showed increased HLA-E surface expression, which impaired CD94/NKG2A-dependent NK cell-mediated cytolysis [152].

#### 2.3.3. HLA-E/NKG2A and Its Role in Cancer Immunotherapy

Given HLA-E’s role in cancer immune escape, it appears as a good candidate to implement the immune checkpoint inhibitory strategy. An ongoing clinical trial using anti-NKG2A monoclonal antibodies in colorectal cancer demonstrates that blocking HLA-E overexpression shows promising results (NCT02980146) (HLA-E CCR). Other studies have also been conducted on HLA-E and its regulatory effects on NKG2A-mediated function to control survival in lung cancer and lymphoma. Monalizumab, an anti-NKG2A blocking antibody, has been used to restore NK and CD8 T cell cytotoxicity. A recent study has revealed that monalizumab can potentiate anti-PD-1/PD-L1 inhibition as a combination therapy [17,153]. The study has demonstrated that monalizumab targets different key aspects of the immune response. It enhances the antitumor activities of both T and NK cells, by blocking the inhibitory function of NKG2A. This also blocks the NKG2A ligand, HLA-E, which is overexpressed in the human tumor microenvironment (TME) and which reduces lymphocyte expression in the TME. Additionally, monalizumab is also well tolerated in humans and has shown promising efficacy results in clinical trials. This evaluation supports its use in combination with targeted therapies, e.g., with cetuximab (an anti-EGFR antibody) in squamous cell carcinoma of the head and neck (HNSCC), whereas a 30% objective response rate (ORRs) was observed. This combination works most likely by the ADCC of NKG2A-expressing NK cells and not by NKG2A-expressing CD8+ T cells [16]. Clinical trials are ongoing using Ibrutinib in chronic lymphocytic leukemia (NCT02557516) [154], trastuzumab in breast cancer (NCT04307329) [155]), durvalumab in colorectal cancer (NCT02671435), and with cetuximab in HNSCC (NCT02643550) [156].

An elegant study showed that PD-1 blockade did not work as an efficient vaccination therapy in mouse tumor models, whereas its combination with NKG2A blockade showed a promising effect. This proves that NKG2A and PD-1 blockade may target a different subset of T cells [157]. Although PD-1/PD-L1 blockade therapy is the first line of treatment for different cancer types, the combination with NKG2A mAb was also tested in the PD-1/PD-L1-responsive MC38 mouse colon tumor model. The results did not show any significant improvement in therapeutic efficacy in the presence of NKG2A mAb, whereas a synthetic peptide vaccine did show an improvement. This result suggests that the therapeutic synergy between NKG2A and PD-1/PD-L1 may involve an overlapping subset of intratumoral CD8+ T cells and this makes NK cells unessential [153] (for more key clinical studies, please refer to Table 1).

### 2.4. NKG2D as an Immune Checkpoint Target

#### 2.4.1. Relationship between Tumor Microenvironment and NKG2D

NKG2D is another receptor of the NKG2 family and is expressed on immune cells of the cytotoxic type [139]. Although NKG2D is expressed less abundantly, it can be induced during the stress response, such as infection and oncogenic transformation, senescent, and stress conditions [158]. NKG2D expression is regulated in different stages, such as transcription, mRNA and protein stabilization, and cleavage from the cell surface via various stress pathways [158]. The NKG2D ligand (NKG2DL) can also be regulated by transcriptional, RNA splicing, posttranscriptional, and posttranslational events [159]. During transcription, NKG2D ligands can be regulated by transcription factors or regulatory sequences in various molecular pathways. Under homeostatic conditions, the expression of NKG2DL is generally low [160]. The regulation of NKG2DL can also be influenced by cell stress, proliferation signals, infection, and oxidative stress, which might activate the DNA damage response or oncogenic transformation [160].

#### 2.4.2. NKG2D: Its Overall Function and Structural Configuration

In NK cells, NKG2D acts as an activating receptor, which triggers cytotoxicity and also functions on CD8+ T cells to generate activating co-stimulatory signals [161] (Figure 1). The unique molecular structure of NKG2D enables it to interact with several structurally different MHCI-like ligands [158,162]. Additionally, in cancer cells, stress conditions upregulate NKG2D expression, which induces NK-cell-mediated lysis. The cancer cells develop a unique mechanism to reduce and eliminate the overexpressed NKG2DL on their surfaces by secreting metalloproteases capable of cleaving these ligands. Soluble NKG2DLs act as decoy molecules that mediate the immunosuppression and tumor immune escape by controlling NK-cell-mediated cytotoxic activity [163]. Cellular senescence induction occurs due to DNA damage mechanisms and upregulates the expression of NKG2D, which enables the NK-mediated killing of senescent cells via the granule exocytosis pathway [164,165].

The impact of NKG2D on NK cell development also depends on the interaction with the interleukin-15 receptor (IL-15R). It has been demonstrated that this IL-15R signaling is important for the development, homeostasis, and survival of NK cells. Studies have revealed that NKG2D-deficient NK cells are prone to apoptosis, which cannot be compensated by the addition of IL-15, suggesting that these two receptors have a common signaling pathway [166].

It has been demonstrated that some tumors downregulate NKG2DL’s expression to prevent its recognition. On the other hand, it has also been shown that some tumors induce NKG2DL expression, and this high NKG2DL expression triggers the downregulation of the NKG2D receptor on NK cells, which reduces their responsiveness [162,167]. This process prevents NK cells’ hyper-responsiveness against NKG2D ligands. Manipulation of the NKG2D–NKG2DL interaction might be a promising immunotherapeutic strategy for the treatment of various cancer types [139].

#### 2.4.3. NKG2D and Its Role in Cancer Immunotherapy

NKG2D is one of the most well-characterized activating immunoreceptors that is associated with tumor immunosurveillance. NKG2D is also an important player in antitumor immunity due to its ability to recognize tumor cells and initiate an antitumor immune response [168].

It has also been reported that chimeric NKG2D-expressing T cells can be potential immunotherapeutics for gastric cancers with peritoneal metastasis [169,170]. Furthermore, the subpopulations of self-enriched repurposed NKG2D CAR T cells induce antitumor cytotoxicity against triple-negative breast cancer growth. Additionally, pre-clinical studies using these NKG2D CAR T cells targeted the NKG2D ligands on cancer cells, which promoted the expression of CD27 and 4-1BB co-stimulatory molecules on these CAR T cells. This resulted in a decrease in tumor burden in vivo [171,172]. It has been reported that NKG2D can be silenced by DNA methylation during the development of acute myeloid leukemia, and NKG2D ligand levels can be increased by using demethylating factors (e.g., azacitidine, decitabine) [173,174], which enhances the cytotoxic effect of the NK cells, resulting in the detection and lysis of cancer cells [172,175]. It has also been demonstrated that autologous activated and expanded NK cells (NKAE) bearing NKG2D-CAR can be used to treat multiple myeloma, and CAR-NKAE cells are a better strategy against MM than memory CAR-T cells [176]. Another pre-clinical study has demonstrated that the monotherapy of B10G5 (sMIC-neutralizing antibody) or combination with ALT-803 (immunostimulatory IL-15 super-agonist complex) significantly increased the NKG2D + CD8+ T cells and decreased the primary tumor burden and abolished metastasis in mouse-bearing B16-sMICB tumors. A significant synergistic therapeutic effect with ALT-803 was also reported to boost the tumor response to anti-CTLA-4 checkpoint blockade therapy [177].

Among many selective inhibitors, only Periostat, also known as Doxycycline (an inhibitor of matrix metalloproteinases (MMPs) that cleaves the extracellular domain of MICA/B, ligands for the NKG2D receptor), is undergoing clinical trials for different types of tumors. Periostat helps to control the increase in NKG2DL cell surface expression, thereby enhancing the NK cell-mediated cytotoxicity [178,179,180]. Some drugs, such as histone deacetylase (HDAC) inhibitors or bortezomib, along with conventional treatments such as chemotherapy, radiation therapy, or immunotherapies, combined with NKG2D-blockade therapies, have shown the significant upregulation of NKG2D ligands on tumor cells, by activating the NKG2D+ effector cells that eliminate the tumor [159] (for more key clinical studies, please refer to Table 1).

### 2.5. A2AR and A2BR as Immune Checkpoint Targets

#### 2.5.1. Relationship between Tumor Microenvironment and A2AR

The adenosine A2A receptor (A2AR) is a class A, G-protein-coupled receptor (GPCR) that has major affinity to adenosine. The signaling of A2AR is responsible for the activation of the cAMP/PKA pathway through coupling to Gαs family members (i.e., Gαs and Gαolf). The increased cAMP level leads to the activation of PKA and several other downstream targets, which include the transcription factor cAMP response element-binding protein (CREB) [181]. A2ARs are known to be responsible for suppressing immune cells and protecting tissues from inflammation [182]. As part of their regulatory role in the adaptive immune system, A2ARs function similarly to PD-1 and CTLA-4 receptors to suppress the immunologic response. Due to cellular stress, the excess extracellular adenosine binds with A2ARs through the response of the Gs protein-coupled receptor, which in turn accumulates cAMP via the activation of PKA and upregulates the inhibitory cytokine TGF-β and inhibitory receptor PD-1 [183,184].

#### 2.5.2. A2AR: Its Overall Function and Structural Configuration

A2AR, as a member of the GPCR family, contains seven transmembrane alpha helices and an extracellular N-terminus along with an intracellular C-terminus. Adenosine is essential for the synthesis of adenosine triphosphate (ATP), adenine, and adenylate [185]. Extracellular adenosine can signal through a set of GCPRs: A1, A2a, and A3, with higher affinity for adenosine, and A2b, with lower affinity [183,184,186,187,188,189]. A2ARs are known to be widely expressed on the surfaces of most immune cells [190,191,192] (Figure 1).

A phase I study on the A2AR pathway has demonstrated that A2AR has a significant role in immunosuppression. Extracellular adenosine binds to the A2AR on the immune cell surface and activates the cAMP pathway, which in turn inhibits T cell activation and expansion [193]. Studies have demonstrated that adenosine tends to accumulate more often in the solid TME due to the presence of transient or chronic hypoxia [194]. Ohta et al. have highlighted that, in an in vivo tumor model, the pharmacological blockade or genetic deletion of the extracellular adenosine–A2AR–cAMP axis can significantly improve the T cell dysfunction in the TME [195].

The ectoenzyme CD39 is abundantly expressed on tumor-infiltrating immune cells, particularly Tregs, effector T cells, and myeloid cells. Increased CD39+ Tregs have been reported in head and neck squamous cell cancer patients, causing adenosine-mediated immune suppression, which was reversed by A2AR or CD39 blockade [196,197]. The very first study on CD39-deficient mice demonstrated that the inhibition of extracellular adenosine-mediated signal transduction can affect angiogenesis and tumor growth [198]. Another study has also demonstrated that the deletion of CD39 in bone-marrow-derived cells can enhance NK-cell-mediated antitumor immunity and inhibit liver metastasis due to melanoma tumors [199]. It is well established that CD73 (an ectonucleotidase) is an immunoinhibitory protein that plays an important role in tumor growth and metastasis. CD73 is mainly responsible for converting extracellular ATP to immunosuppressive adenosine in concert with CD39 in normal tissues, to control the excessive immune response [200,201]. The study has also revealed that the knockdown of CD73 can increase the survival of tumor-bearing mice and enhance the adoptive T cell therapy [200,202]. In addition to this, another study also showed that anti-CD73 antibody therapy inhibits breast tumor growth and metastasis in a mouse model through adoptive immunity and A2ARs on immune cells [188,203,204]. Phase III clinical studies demonstrate several blocking strategies against the A2AR immune suppression pathways, such as anti-hypoxia agents, anti-CD39 agents, anti-CD73 agents, A2AR/A2BR antagonists, and anti-PKA agents [188,205,206]. Some pre-clinical studies including anti-CD73 antibody, BMS-986179 [207], CPI-006 [208], A2AR antagonist NIR178 (PBF-509) [209], inupadenant (EOS-850) [210], Ciforadenant (CPI-444) [211,212], and A2AR/A2BR dual antagonist AB928 [213,214] showed significant tolerability in humans for cancer treatment [188]. Another study on two breast cancer mouse models (4T1.2 and E0771) showed that anti-CD73 antibody reduced both primary tumors and metastases [203] (for more key clinical studies, please refer to Table 1).

#### 2.5.3. A2AR and Its Role in Cancer Immunotherapy

Recent works demonstrate that the adenosine pathway has been of major clinical interest in cancer immunotherapy [206]. Currently, the A2AR clinical trials are focused on three main points: (1) monotherapy and combination with anti-PD-L1 antibodies showed complete and prolonged inhibition of A2AR, which is well tolerated in cancer patients; (2) A2AR antagonists elicit antitumor immunity from monotherapy and in the combination with anti-PD-L1, and (3) different pools of patients need to be identified based on the predictive biomarkers of adenosine pathway blockade [215]. There is also major interest in exploring A2AR antagonists. For example, clinical trials on ciforadenant or ciforadenant in combination with atezolizumab have been the first to publish clinical results on the safety, efficacy, and biomarkers of A2AR antagonists in renal cell cancer patients, as assessed by Corvus Pharmaceuticals [188]. Some other studies have also demonstrated the efficacy of an A2AR antagonist (AZD4635) in metastatic castration-resistant prostate cancer (mCRPC) patients during an AstraZeneca trial. Some other major ongoing trials using A2AR antagonists are AB928 by Arcus Biosciences, NIR178 by Novartis, and EOS100850 by iTeos Therapeutics [209,212,215,216,217,218,219] (for more key clinical studies, please refer to Table 1).

A phase I clinical trial (NCT02655822) of an A2AR antagonist (PBF-509 and CPI-444) alone or with a PD-L1 inhibitor, atezolizumab, has also been performed to evaluate the clinical efficiency of A2AR blockade for solid tumors, e.g., HNSCC [206]. Another clinical study has demonstrated that a high-affinity and selective A2AR inhibitor, ZM241385, in combination with anti-CTLA-4 mAb inhibited tumor growth and enhanced antitumor immune responses in a B16F10 mouse melanoma model [220,221]. Istradefylline has been recognized as an extremely strong, selective, and orally active A2AR antagonist [222]. SCH58261 has been recognized as a potent, selective, and competitive antagonist of A2AR against melanoma and breast cancer mouse models in combination with anti-PD-1 mAb [223,224,225,226]. Another A2A receptor antagonist, Preladenant (SCH-420814), is also a powerful and competitive antagonist of the human A2AR [188,227]. SYN115, another antagonist, has also been observed to enhance tumor immunotherapy in combination with anti-PD-1 mAb in CD73-expressing tumors [228]. FSPTP (an irreversible inhibitor) is another potent A2AR blocker administered as an intratumoral injection, which reduced the frequency of tumor-infiltrating CD8+ T cells, but not CD4+ T cells or NK cells, in an MB49 bladder cancer model [229]. Various studies have demonstrated that blocking A2AR is a promising tumor immunotherapeutic target via its effect on NK cells, myeloid-derived suppressor cells, and tumor-associated macrophages.

#### 2.5.4. Relationship between Tumor Microenvironment and A2BR

Similar to A2AR, the adenosine A2B receptor (A2BR) is also a G-protein-coupled adenosine receptor. The A2BR integral membrane protein stimulates adenylate cyclase activity in the presence of adenosine and is also responsible for interacting with netrin-1, which is involved in axon elongation [230]. As A2BR has low affinity for binding with adenosine, it is evident that, unlike all other adenosine receptor subtypes, A2BR is only expressed during specific physiological conditions with a high adenosine concentration, and not simply at regular physiological levels [231,232]. This unique feature enables A2BR to be active during specific pathophysiologic conditions associated with massive adenosine release, such as in the TME [233].

#### 2.5.5. A2BR: Its Overall Function and Structural Configuration

The A2BR protein contains a single polypeptide chain that spans the membrane seven times. The amino terminal remains on the extracellular side, while the carboxy terminal remains in the intracellular portion [230].

Studies have shown that some transcriptionally regulated physiological conditions, such as hypoxia-inducible factor (HIF)-1a-associated inflammatory hypoxia, enhance the expression of A2BR, which suggests that A2BR may play a major role in tumor promotion [234]. An in vitro study has demonstrated that A2BR plays an opposing role in cancer cell proliferation and apoptosis based on the concentration of adenosine receptor agonists, the level of A2BR, and the type of cancer cell line. Results show that 5′-(N-ethylcarboxamido) adenosine (NECA, a nonspecific adenosine receptor agonist) treatment activates caspase-3, which induces apoptosis in ovarian cancer cell lines in the presence of high A2BR expression [235]. Another study has also demonstrated that the knockdown of A2BR in mouse and human tumor cell lines plays a critical role in the reduction of metastasis by inducing cell cycle arrest and reducing viability and colony-forming ability [236]. It was reported that knocking down the expression of A2BR inhibited tumor growth and stimulated apoptosis in gastric cancer [237]. Other studies demonstrated that the inhibition of A2BR decreased metastasis in melanoma, breast cancer, and renal cell carcinoma [238,239].

#### 2.5.6. A2BR and Its Role in Cancer Immunotherapy

Many studies have demonstrated that A2BR is highly expressed in many murine and human tumors due to the hypoxic environment prevalent in solid tumors. It is also demonstrated that A2BR plays an important role in the TME by promoting tumor proliferation, tumor angiogenesis, tumor cell invasion, metastasis, and immune suppression. Thus, several strategies are being explored by targeting A2BR, and blocking A2BR seems to be a promising anticancer therapeutic strategy [240,241,242] (Figure 1).

A2BR inhibition by antagonist PSB-1115 was shown to decrease the tumor metastasis of CD73+ melanoma cells and mammary cancer [236]. It has been observed that PSB-1115 delays tumor growth and enhances the antitumor activity of dacarbazine, a drug that is currently used in metastatic melanoma treatment. Several antagonists have been in different phases of clinical trials for cancer treatment, and many of these are mixed A2AR/A2BR antagonists such as AB928 (Phase 1, lung cancer, NCT03846310; Phase 1, breast and ovarian cancer, NCT03719326; Phase 1, gastrointestinal cancer, NCT03720678; Phase 1, advanced cancer, NCT03629756), PBF-1129 (Phase 1, non-small-cell lung cancer, NCT03274479), and theophylline. Seitz et al. showed that AB928 (NCT05024097) is recognized as a safe agent and has also been used in phase 1 clinical trials in healthy volunteers. Additionally, it has also been evaluated in patients with non-small-cell lung cancer, breast cancer, ovarian cancer, colorectal, and six other types of cancer [213]. A recent study on rectal cancer showed that AB122, a human PD-1 inhibitor, administered together with AB928 (NCT05024097), was well tolerated and demonstrated evidence of a clinical benefit, including an antitumor response and disease stabilization for more than 6 months [213,242,243]. AB928 used in conjunction with AR inhibition alleviated adenosine-mediated immune suppression. It was demonstrated that AB928 and chemotherapy together result in greater immune activation and tumor control [244]. Another phase 1 clinical study on A2BAR antagonist PBF-1129 in patients with advanced NSCLC, in a dose escalation manner, showed significant tolerability [242]. The study reported that theophylline, a nonselective AR antagonist, may block all four ARs. Theophylline in combination with prednisone and dextromethorphan has also been undergoing a phase 1 clinical trial (NCT01017939) for patients with metastatic castration-resistant prostate cancer. Aminophylline, a salt of theophylline, in combination with Bacillus Calmette–Guerin, has been in an early phase 1 trial for patients with bladder cancer (NCT01240824) [242] (for more key clinical studies, please refer to Table 1).

### 2.6. SIRPα/CD47 as an Immune Checkpoint Target

Increasing studies report that targeting signal regulatory protein alpha (SIRPα)/CD47 results in therapeutic success in cancer treatment. In physiological conditions, the SIRPα/CD47 pathway is involved in immunotolerance; however, in malignancy, it helps the cancer cells to achieve immune evasion. The SIRPα/CD47 binding inhibits the antitumor immune response, which makes this a crucial druggable target in cancer immunotherapy. To avoid this binding action and allow the clearance of tumor cells, many blocking antibodies have been developed [245,246,247].

#### 2.6.1. SIRPα/CD47 Functions

SIRPα, also known as SHPS-1 or CD172a, is expressed in myeloid cells, including monocytes, macrophages, granulocytes, and CD4+ DCs, as well as in neurons [248]. In humans, as a transmembrane protein, it possesses (a) an extracellular domain with three Ig-like regions (NH2-terminal immunoglobulin variable region and two Ig constant regions), (b) a transmembrane domain, and (c) an intracytoplasmic region with two immunoreceptor tyrosine-based inhibitory motifs and a proline-rich region, which binds Src homology domain-containing proteins [249,250].

CD47, known as integrin-related protein (IAP), is broadly expressed on all cell types, including tumor cells [251]. It is also a transmembrane protein that has (a) an extracellular NH2-terminal Ig variable-like domain, (b) a 5-transmembrane spanning helical bundle domain, and (c) a cytoplasmic COOH-terminal domain. It functionally binds αVβ3 integrin, SIRPα, and thrombospondin-1, resulting in the activation of several homeostatic signaling pathways that regulate cell proliferation, differentiation, migration, angiogenesis, and host defense as well as the immune response [252,253]. CD47 is known as a “do not eat me” signal that, following its binding with SIRPα on macrophages, prevents the macrophage-mediated phagocytosis that represents the engulfment of tumor cells by phagocytosis-specialized macrophages [254,255].

CD47, with its NH2-terminal IgV domain, binds SIRPα. This binding generates the phosphorylation of tyrosine residues that in turn binds and activates protein tyrosine phosphatases (PTPase), such as SHP1 and 2, which deactivates the autoinhibitory activity and favors enzymatic activity, including the limitation of phagocytosis [256].

#### 2.6.2. SIRPα/CD47 Role in Cancer Immunosuppression

In 2000, CD47 was identified on red blood cells as a marker of self-cells. Indeed, CD47 binds SIRPα on macrophages, therefore recognizing the red blood cells as self and not eliminating them in the spleen [257]. The SIRPα/CD47 homeostatic function also occurs in the context of cancer to escape the immune system. An elegant work by Jaiswal et al. shows that the upregulation of CD47 on hematopoietic stem cells and leukemia cells protects them from phagocytosis, allowing mobilization and increasing pathogenicity, respectively [258]. Indeed, the blocking antibody against SIRPα on macrophages favors the phagocytosis of CD47-expressing cancer cells [258]. In parallel, the role of the SIRPα/CD47 axis has been described in head and neck squamous cell carcinoma (HNSCC). CD47 expression is increased in human and murine HNSCC. Furthermore, it correlates with poor prognosis in patients. Moreover, CD47 is associated with immune checkpoint proteins, such as PD-1 and PD-L1, the Treg marker Foxp3, and the immunosuppressive myeloid-derived suppressor cell (MDSC) markers, including CD11b and CD33 [259]. Treatment with a monoclonal anti-CD47 antibody delays the tumor growth in HNSCC in immunocompetent mice, affecting the AKT pathway [259]. Importantly, anti-CD47 treatment induces a switch from an immunosuppressive to immunogenic microenvironment by decreasing PD-1 expression and increasing IFN-γ secretion by T effector cells, as well as reducing the suppressive function of CD11b+ Ly6G+ Ly6Clo MDSCs in vivo [259]. In non-Hodgkin’s lymphoma (NHL), CD47 is required for NHL cell extranodal dissemination, which was inhibited by a CD47 blocking antibody [260]. Anti-CD47 treatment is also effective in glioblastoma by stimulating M1 macrophage-dependent phagocytosis in vivo [261] (Figure 1).

Nowadays, SIRPα/CD47 is inhibited in combination with other checkpoint immunotherapies to treat multiple types of cancer. The dual anti-CD47/anti-PD-L1 blockade successfully reduces murine colorectal tumor growth via the activation of dendritic cells and macrophages and differentiation of Tcf7+ stem-like progenitor CD8+ T cells in T effector cells [262]. Similarly, CTLA-4 × SIRPα antibody, which targets both CTLA-4 and CD47 on ICOS-high T regulatory cells, enhances antitumor immunity in murine colon cancers [263]. Furthermore, in esophageal squamous carcinoma, anti-CD47 treatment favors the expression of pro-inflammatory cytokines (IL-2 and IL-12, IFN-γ, TNF-*β*) and increases the tumor-infiltrating CD8+ T cells [264]. Importantly, these cells express high levels of immune checkpoints PD-1 and CTLA-4, suggesting their activated status, indicating that anti-CD47 treatment induces an antitumor immune response [264]. Therefore, the synergic combination of anti-CD47 with anti-PD-1 and anti-CTLA-4 induces a delay in tumor growth and improves overall survival in mice. Moreover, CD47 is highly expressed in patients with low tumor CD8+ T cell infiltration, confirming its negative role in the antitumor immune response [264]. Many clinical trials have been developed using anti-CD47 (NCT02641002) or bifunctional CD47-PD-1 antibodies (NCT04886271), or anti-SIRPα antibodies (NCT02663518, NCT0399023), with promising and hopeful results for several types of cancer (for more details, please refer to Table 1).

Overall, SIRPα/CD47 blockade results in a therapeutic strategy for cancer treatment that involves macrophages as key players in tumor immune escape.

### 2.7. TIM-3 as an Immune Checkpoint Target

Along with the well-known PD-1, PD-L1, and CTLA-4, and in addition to SIRPα/CD47, TIM-3 has emerged as a promising target in cancer immunotherapy [265].

#### 2.7.1. Role of TIM-3

T cell immunoglobulin and mucin domain 3 (TIM-3), also known as hepatitis A virus cellular receptor 2 (HAVCR2), a member of the TIM family, is a membrane glycoprotein expressed by monocytes, macrophages, mast cells, NK, DCs, B cells, and T cells, including Th1 cells, Th17 cells, Tregs, and type 1 CD8+ T cells (Tc1) [266,267]. Structurally, TIM-3 is composed of an N-terminal extracellular IgV domain, a transmembrane domain, and an intracellular domain with four phosphor-tyrosine domains. TIM-3 has several ligands and different functions depending on which ligand it binds. Galectin-9 (Gal-9) binds TIM-3 expressed by T cells inducing Ca(2+)-calpain-caspase-1 driven apoptosis of TIM-3+ T cells [268] and inhibits the cell proliferation and secretion of TNF-α and IFN-γ [269,270]. When TIM-3 is expressed by macrophages and T cells, it binds Gal-9, with inhibitory effects on the antitumor response [271]. The TIM-3/Gal 9 complex inhibits the proliferation of T cells and decreases the secretion of cytokines (IFN-γ, TNF-α, and IL-2), which results in the death of T cells. In Tregs, the interaction of TIM-3/Gal-9 promotes immunosuppressive TGF-*β* [272] and interleukin-10 (IL-10) signaling [273]. On the contrary, when it is expressed by DCs and NK cells, it has activating functions [271,274]. High Mobility Group Box 1 (HMGB1) binds TIM-3 expressed by DCs, blocking the endosome trafficking of the nucleic acids released by apoptotic cells, inhibiting the pattern recognition receptor (PRR) signaling [275]. On the other hand, the interaction between TIM-3 expressed by T cells and HMGB1 is not fully understood. Moreover, TIM-3 also binds phosphatidylserine (Ptdser), exposed on the outer surface of the cellular plasma membrane by apoptotic cells, as a signal to undergo phagocytosis [267]. TIM-3+ CD8+ DCs binds Ptdser and contributes to apoptotic cell clearance; indeed, anti-TIM-3 antibody inhibits the cross-presentation of antigens derived by apoptotic cells [276].

#### 2.7.2. TIM-3 in Cancer Immune Escape and Clinical Applications of TIM-3 Antibodies

Solid tumors, including breast, head and neck, prostate, NSCLC, and glioma [267,277], express high levels of TIM-3 on T cells and Gal-9 on cancer cells, rendering TIM-3/Gal-9 binding a target of immunotherapy. TIM-3 is co-expressed with PD-1 in terminally differentiated T cells. Currently, many TIM-3 inhibitors have been developed for monotherapy or combination therapy for cancer management. Several clinical trials are using TIM-3 antibodies [266], including Sym023 (NCT033114112), ICAGN02390 (NCT03652077), TSR-022 (NCT02817633), MBG453 (NCT02608268), LY3321367 (NCT03099109), BGBA425 (NCT03744468), R07121661 (NCT03708328) [278], and BMS-986258 (NCT03446040). The combination of anti-TIM-3 sabatolimab with anti-PD-1 spartalizumab shows promising antitumor activity in several solid tumors [279] (for more key clinical studies, please refer to Table 1).

### 2.8. LAG-3 as an Immune Checkpoint Target

Another member of the superfamily of immunoglobulins that also is a significant potential ICB is lymphocyte activation gene-3 (LAG-3), also known as CD223 [280,281].

#### 2.8.1. Function of LAG-3

LAG-3 is a surface receptor on TILs [282], activated CD4 [283] and CD8 T cells [284], Tregs [285,286], NK cells, DCs [287,288], and B cells [289]. As a surface receptor, LAG-3’s structure is composed of (a) an extracellular domain with D1, D2, D3, and D4 immunoglobulin superfamily domains that bind the ligands; (b) an intramembrane domain; and (c) intracellular domains with the serine phosphorylation site S454 bound by protein kinase C, the “KIEELE” motif crucial for LAG-3 function, and the EP sequence that is a glutamate–proline dipeptide repeat motif [290]

The extracellular domain binds the LAG-3 ligands, which are galactose lectin-3 (galectin-3), MHCII, fibrinogen-like protein 1 (FGL1), and hepatic sinusoid endothelial cell lectin (LSECtin).

Galectin-3 is a soluble lectin that is ubiquitously expressed [291], whereas LAG-3 is glycosylated, and it binds galectin-3, inhibiting the CD8+ T cell production of IFN-γ [292].

LAG-3, as well as CD4—as they have structural high homology—binds MHCII but at a higher affinity than CD4 [293]. This interaction induces the inhibition of TCR signal transduction [294,295] and ITAM-driven DC activation [296].

FGL1 is expressed by tumor cells with different cellular localizations. Indeed, it has been described to be present both on the surfaces of breast cancer cells and in the cytoplasm of NSCLC cells [297,298]. It is not clear how FGL1 from the cytoplasm can interact with LAG-3 on the surfaces of lymphocytes, or whether other molecules are involved as mediators of the ligand/receptor interaction. The downstream signaling of FGL1/LAG-3 is unknown. However, upon interaction, a decrease in IL-2 secretion occurs, leading to the inhibition of the antigen-mediated T cell response [297].

LSECtin is a member of the C-type lectin receptor superfamily, and it is expressed in the liver, lymph nodes [299], and melanoma [300]. Its binding with LAG-3 determines the suppression of the antitumor T cell response with the promotion of tumor growth [300].

The signaling transduction of LAG-3 is not well defined. The motif “KIEELE”, interacting with unknown binding partner molecules, transmits the signal, leading to the cell cycle arrest of T cells with a subsequent loss of T cell expansion [301,302]. Moreover, Previte and colleagues demonstrated that LAG-3 on naïve CD4 T cells negatively modulates mitochondrial biogenesis and homeostatic cell expansion through the STAT5 and Akt pathways to maintain the cells in a quiescent status [303]. Furthermore, LAG-3 is expressed on CD4 (+) CD25 (high) Foxp3 (+) T cells, which represent an expanded cell subset that secretes immunosuppressive cytokines such as IL-10 and TGF-*β*1 in patients with melanoma and colorectal cancer [304].

LAG-3 expression on the cell surface is regulated by endocytosis [305] or by the activity of the metalloproteases ADAM10 and ADAM17 that cleave LAG-3, generating a soluble protein, sLAG-3 [306].

LAG-3 is overexpressed in many tumors, including melanoma, gastric, ovarian, anal and colorectal, hepatocellular, prostate, follicular, breast, head and neck, NSCLC, renal, pancreatic, and mesothelioma cancer [281] (Figure 1).

#### 2.8.2. Clinical Application of LAG-3 Blocking Antibodies

Many clinical applications to inhibit the LAG-3/ligand binding have been developed for cancer treatment. The prevention of binding between LAG-3 with MHCII with a cyclic peptide, C25, increases IFN-γ secretion and CD8 T cell infiltration and decreases the FOXP3+ Tregs in both in vivo colon–rectal and melanoma models [307]. Similarly, Leramilimab (LAG525), another inhibitor of the LAG-3/MHCII interaction, has antitumor activity in combination with anti-PD-1 spartalizumab in advanced/metastatic solid tumors (NCT02460224). In chronic lymphocytic leukemia, relatlimab, a monoclonal anti-LAG-3 antibody, recovers the activity of T and NK cells, promoting the antitumor immune response [308] (NCT03470922) (for more key clinical studies, please refer to Table 1). Further studies are required to better characterize the LAG-3 interaction with its ligands, especially FGL-1, and dissect the downstream signaling to improve its inhibition and promote the antitumor immune response.

### 2.9. B7-H3 as Immune Checkpoint Target in Cancer

Among the new tumor antigens that are emerging as potential targets for cancer immunotherapy, B7-H3 is being reported as a powerful checkpoint target [309].

#### 2.9.1. Role of B7-H3/CD276 in Immune Response

Activated APCs express B7 on their membranes [309]. The B7-H3 subvariant protein (or CD276) was overexpressed in different types of human cancer cells and was associated with disease deterioration [310,311,312]. B7-H3 usually functions as a co-stimulatory receptor required for immune responses such as the stimulation of T cells and IFN-γ expression [310] (Figure 1).

#### 2.9.2. Role of B7-H3/CD276 in Immune Suppression in Tumors

Recently, it was observed that B7-H3 can exert inhibitory effects toward T cell proliferation and triggers enhanced immunosuppression [312,313,314]. Studies show that B7-H3 hinders the proliferation of both CD4 and CD8 T cells [315]. Other work suggested that B7-H3 overexpression was positively correlated with a greater tumor load, advanced clinical stage, and low survival percentages in oral squamous cell carcinoma patients. It was also shown that tumor cell proliferation was inhibited by B7-H3 blockade, while the rescue of B7-H3 expression enhanced tumor proliferation [316]. B7-H3 and Tregs were found to have a possible collaborative role in tumor immune escape and the subsequent poor outcomes in NSCLC patients [317]. B7-H3 and CD14 were co-expressed in renal cell carcinoma tissue, which was positively correlated with an increased tumor burden. This correlation points towards the significant function of B7-H3 in CD14+ monocyte-mediated RCC angiogenesis [318]. Similarly, another study suggested that the co-expression of B7-H3 and CD133 correlated with disease progression in CD133+ CRC (colorectal cancer) [319]. The B7-H3 overexpression observed in human breast cancer tissues may play a vital role in tumor growth and invasiveness via the enhanced secretion of the immunosuppressive cytokine IL-10 [320]. Several other reports suggest the role of B7-H3 in drug resistance during tumor therapy via the upregulation and activation of different pro-tumoral signaling cascades [309].

Inspired by pre-clinical studies, strategies to block B7-H3 have been developed and have been tested in cancer patients in clinical studies [309]. Notable examples include Enoblituzumab (MGA271), a humanized monoclonal antibody against B7-H3. Enoblituzumab facilitated potent antibody-dependent cellular cytotoxicity (ADCC) against a broad range of tumors [321]. Several trials, such as NCT02923180/Phase 2 and NCT04145622/Phase 1, are ongoing using MGA271. Studies have also been conducted using activated T cells (ATC) bearing anti-CD3 x anti-B7-H3 moieties (referred to as B7-H3Bi-Ab), which showed effective cytotoxicity towards tumor cells by ADCC. ATC possessing B7-H3Bi-Ab showed augmented cytotoxic effects, enhanced cytokine expression, inhibition of the tumor burden in vivo, and significantly improved survival [322]. Combination trials using anti B7-H3 +/− anti PD-1 on a broad spectrum of cancers are ongoing (NCT03729596/Phase 1, 2). Evidence from the above studies, along with several others, indicates B7-H3 as a promising checkpoint target in different tumors that is worth further exploration (for more key clinical studies, please refer to Table 1).

### 2.10. PARPs as Promising Immune Checkpoint Targets in Cancer

Poly ADP-ribose polymerases (PARPs) play a pivotal role in both innate and adaptive immune responses. Given that T cells are the principal immune cells involved in antitumor immunity, PARP inhibition significantly impacts T cell functions in the TME [323].

#### 2.10.1. Functions of PARPs in DNA Damage Response

PARP enzymes, including PARP1 and PARP2, are vital DNA damage detecting and signaling proteins involved in the DNA damage response. Cells are persistently faced with external and internal stresses that can ultimately trigger DNA damage. To maintain genomic stability and prevent carcinogenesis, the detection of DNA damage and subsequent DNA repair are vital biological processes that are managed by multiple pathways in cells [324]. These enzymes bind to DNA breaks and catalyze the attachment of poly (ADP-ribose) (PAR) moieties on target proteins (referred to as PARylation) in the vicinity of the DNA break and also on themselves (self-PARylation) [325,326]. These negatively charged PARylated chains stimulate posttranslational modification-mediated cellular effects such as chromatin remodeling and the recruitment of DNA repair protein complexes and also influence the pace of replication fork progression [323,327]. The binding of PARP1 through zinc finger domains to sites of DNA damage triggers a change in conformation in the PARP1 proteins and alleviates the self-repressive interaction between the catalytic domain and helical domain. Subsequently, PARP1 utilizes nicotinamide (β-NAD+) as its substrate to catalyze the transfer of ADP-ribose chains onto target proteins. This posttranslational modification of the PARylation of target proteins most likely mediates DNA repair [328,329] (Figure 1).

#### 2.10.2. Antitumor Role of PARP Inhibitors (PARPi) in Cancer in the Context of the Tumor Microenvironment (TME)

Besides their usual functions in maintaining genomic integrity, multiple studies have shown that PARPs are involved in anti-cancer immunity. In small cell lung cancer (SCLC), PARPi was demonstrated to trigger the activation of cytotoxic T lymphocytes (CTL) via upregulating the STING/TBK1/IRF3 innate immune pathway, and to increase the expression of chemokines such as C-X-C motif chemokine ligand 10 (CXCL10) and C-C motif chemokine ligand 5 (CCL5) [330]. Studies in ovarian cancer showed that PARPi could stimulate an increase in PD-L1 expression via the enhanced phosphorylation of CHK1. As a result, PD-L1 blockade and PARPi caused a synergistic antitumor immune response via CD8+ T cells [331]. Therapeutic studies with Talazoparib (BMN673, a PARP1/2 inhibitor) significantly increased the frequency of activated NK cells with the enhanced secretion of IFN-γ and TNF-α in the TME of a murine ovarian cancer model [332]. Other pre-clinical cancer studies also demonstrated that the inhibition of PARP-1/2 retained NK cell viability and primed tumor cells to NK-cell-mediated killing in various cancer models, such as breast, prostate, NSLC, and chronic myeloid leukemia [333,334].

PARPi has been shown to be an efficacious therapeutic strategy against cancers with defects in double-strand break (DSB) repair. Recently, various PARPi have been used in more than 70 clinical trials and have been approved by the FDA, including Olaparib, niraparib, rucaparib, and talazoparib [335,336,337,338,339]. Olaparib was recently approved for first-line maintenance treatment in BRCA1/2-mutated, newly diagnosed, advanced ovarian cancer after a complete response (CR)/partial response (PR) to platinum-based chemotherapy [340]. PARPi-associated clinical studies were performed in various cancer types, including ovarian cancer, fallopian tube cancer, primary peritoneal cancer, high-grade endometrioid cancer, breast cancer, pancreatic cancer, prostate cancer, and lung cancer [336]. For example, phase 2 trials are ongoing (NCT00753545) using PARP inhibitor Olaparib in patients with ovarian cancer that recurred within 12 months of prior platinum therapy, with confirmed germline BRCA1/BRCA2 mutation. A phase 1 trial (NCT03101280) is also ongoing using PARP inhibitor rucaparib in advanced gynecologic cancer and triple-negative breast cancer patients. Combination therapies based on a synergistic antitumor effect due to PARPi and other antitumor therapeutics warrant further exploration based on the promising pieces of evidence mentioned earlier. Initial studies of PARPi-related combined therapeutics mostly focused on chemotherapy, radiotherapy, and a few other standard target regimens. Recent studies have shown that PARP inhibition can enhance the responses of other ICIs [341,342]. PARPi treatment led to an increase in DNA damage and triggered the interferon pathways. Thus, PARPi has the potential to improve the response to ICIs by enhancing the T-cell-mediated immune response [324,343]. Promising results from pre-clinical studies of PARPi in combination with ICIs have been translated into clinical trials, which in turn have also shown encouraging results [324] (for more key clinical studies, please refer to Table 1).

### 2.11. TIGIT as a Promising Immune Checkpoint Target in Cancer

In addition to the previously mentioned immune checkpoint targets, other, newer targets are gaining prominence in pre-clinical and clinical studies. T cell immunoreceptor with immunoglobulin and ITIM domain (TIGIT, also called WUCAM, Vstm3, VSIG9) is becoming a promising ICI target. TIGIT expression is elevated by different immune cell populations, such as activated T, NK, and Treg cells [344] (Figure 1).

#### 2.11.1. Structure and Functions of TIGIT in Immune Cells

TIGIT is a receptor of the Ig superfamily. TIGIT is composed of (i) an Ig variable domain that is extracellular, (ii) a type 1 domain that is transmembrane, and (iii) two inhibitory motifs (conserved in mouse and human): an ITIM and an Ig tail-tyrosine (ITT)-like motif, which is the cytoplasmic tail [345,346,347,348].

TIGIT functions as a negative regulator of adaptive and innate immune cells. TIGIT plays important roles in regulating the functions of signaling cascades involving multiple immune inhibitory receptors, such as CD96/TACTILE and CD112R/PVRIG, a competing co-stimulatory receptor DNAM-1/CD226, and multiple ligands, e.g., CD112 (Nectin-2/PVRL2) [346,347,349,350,351]. TIGIT can downregulate the immune response in different ways. For example, TIGIT indirectly inhibits T cell function by engaging with the CD155 receptor expressed on DCs. This binding stimulates CD155 phosphorylation and activates a signaling cascade that enhances the tolerance phenotype in these DCs. This causes the DCs to produce less IL-12 and more IL-10 to trigger immunosuppression [347]. Additionally, TIGIT has other multiple mechanisms by which it suppresses the activity of other immune cells such as T cells and NK cells [344].

#### 2.11.2. Role of TIGIT in Immune Suppression in the TME

A study showed that sole TIGIT blockade or TIGIT/PD-1 blockade decreased the tumor burden through NK cell activation, which enhanced CD8 T cell-mediated antitumor immune responses in B16 melanoma and CT26 lung metastatic mouse models [352]. Blockade of TIGIT and other immune checkpoint targets as cancer immunotherapeutic strategies has been reported to be very promising in different pre-clinical mouse tumor models. In a mouse colorectal cancer model, individual single blockade failed to significantly inhibit tumor growth. In sharp contrast, blocking TIGIT and PD-1/PD-L1 gave highly synergistic results, including absolute tumor rejection with enhanced survival, and enhanced activation, memory, and antitumor functions of CD8 T cells. Additionally, all of the above effects were dampened upon CD8 T cell depletion, signifying the important immunoinhibitory role of TIGIT in CD8 T cell antitumor functioning [352,353,354]. Blockade of TIGIT and TIM-3 synergized to enhance antitumor immune responses in various mouse tumor models via Treg cells [355].

The potential of TIGIT as a credible cancer immunotherapeutic target is being tested in multiple phase I and II clinical trials. Most of these trials are testing Fc-engineered anti-TIGIT mAbs. One such study is testing TIGIT blocking human IgG1 mAb in a phase I/II study (BMS-986207/Bristol-Myers Squibb) with other ICIs such as LAG-3 in multiple myeloma patients with relapse (NCT04570839). Another example is a phase I study of TIGIT blocking humanized IgG1 mAb BGB-A1217 (BeiGene) in patients with metastatic solid tumors (NCT04047862). Details of these and other clinical trials using TIGIT inhibition are mentioned in Table 1.

### 2.12. VISTA as a Target of Cancer Immunotherapy

Recently, another molecule of interest has emerged in immunotherapy research called the V-domain Ig suppressor of T cell activation (VISTA, also named c10orf54, VSIR, SISP1, B7-H5, PD-1H, DD1α, Gi24, and Dies1). VISTA, a B7 family member, is an immunoglobulin superfamily inhibitory ligand expressed on myeloid cells, including microglia, neutrophils, monocytes, macrophages, CD11b+ CD8+ T cells, and naïve CD4+ and Foxp3+ Treg cells [356]. VISTA is also expressed in cancer cells, including epithelioid malignant pleural mesothelioma [357], melanoma [358], lung [359], breast [360], renal [361], colorectal [362], and gynecologic cancers [363].

#### 2.12.1. VISTA’s Structure and Functions

Structurally, VISTA, as a transmembrane protein, consists of (1) an N-terminal extracellular Ig domain that shares homology with the B7 family ligands (highest homology with PD-L1, 22%). The VISTA extracellular domain exclusively contains four cysteines not found in the other family members. It also contains (2) a transmembrane domain and (3) a cytoplasmic tail without ITIM and ITAM motifs, differing from the other B7 molecules. However, it has protein kinase C binding sites and a proline-rich motif for protein–protein interactions for signal transduction [364]. It is unclear whether VISTA acts as a receptor, ligand, or both. The known VISTA ligands are V-set and Ig domain-containing 3 (VSIG3, expressed on colorectal cancers, hepatocellular carcinomas, and intestinal-type gastric cancers) and, at an acidic pH, such as in the TME, P-selectin glycoprotein ligand 1 (PSGL-1). It also binds the lesser known VSIG8. However, further studies are required to clarify the VISTA/ligand pathways [365] (Figure 1). Physiologically, VISTA contributes in maintaining cell and myeloid quiescence. The ligand binding induces immune suppression. Indeed, VISTA inhibits T cell activation by TCR phosphorylation. Furthermore, VISTA expressed by APCs binds the coinhibitory receptor on T cells, inducing their suppression [357,365].

#### 2.12.2. VISTA, a Potential Target for Cancer Immunotherapy

Recently, VISTA was demonstrated as a potential and powerful immune checkpoint. JNJ-61610588, a human IgG1 mAb against VISTA, was the first antibody tested in a phase I trial (NCT02671955) in patients with solid advanced tumors (lung, pancreas, cervical, colorectal, head and neck), whose data analysis is still ongoing. Similarly, patients with relapsed/refractory solid tumors are being recruited for another phase I clinical trial (NCT04475523) to test CI-8993, a human IgG1_k_ monoclonal anti-VISTA ligand antibody. Moreover, combination therapies blocking VISTA and PD-1 or VISTA and PD-L1/2 have been tested to improve T cell functionality and reduce tumor growth. For instance, CA-170, which targets VISTA and PD-L1/2, increased T cell activation and cytokine secretion in a phase I trial conducted in patients with advanced solid cancers and lymphoma (NCT02812875). Detailed information and other clinical trials are listed in Table 1.

### 2.13. mARTs as Promising Immune Checkpoint Targets in Cancer

Mono-ADP-ribosyl transferases (mARTs) utilize NAD+ as a substrate and transfer mono-ADP-ribosyl (MAR) moieties to target proteins to modify cellular functions posttranslationally. They are related to the PARPs, but most of them, unlike PARPs, have a presence on the cell surface [366]. Pioneering work from Wennerberg and Mukherjee et. al. has recently shown a novel role of tumor cells expressing mono-ADP-ribosyl transferase 1 (ART1) in triggering immunosuppression in pre-clinical mouse models of NSCLC and melanoma. These studies showed that ART1 expressed by tumor cells caused NAD+-induced mono-ADP ribosylation (MARylation) and the subsequent cell death (NICD − NAD+-induced cell death) of cytotoxic CD8 T cells and DCs to enhance the tumor burden in different murine tumor models. Interestingly, inhibiting ART1 expression by genetic ablation or blocking ART1’s enzymatic activity using a humanized monoclonal activity triggered a robust antitumor immune response, which resulted in a significant reduction in tumor burden [367,368,369,370]. Further studies are warranted in other tumor models to study the role of ART1 as a regulator of immunosuppression in the TME. Additionally, combination studies involving ART1 inhibitors with ICIs in pre-clinical cancer models will be important to determine their synergism in therapy (Figure 1).

## 3. Discussion

Despite many ICIs approved for clinical use and others undergoing clinical trials, there are still major challenges. The repertoire of ICIs needs to be increased so that patients with different cancer types positively respond to them. Additionally, we need to focus on overcoming the acquired resistance to ICI therapy. The success of a PD-L1 blocker depends on the relative expression of PD-L1 in a variety of tumor cells. Cancers with lower levels of expression are therefore poor responders to these therapeutics. This situation warrants the identification of biomarkers to personalize treatment and achieve better outcomes. Researchers have realized with time that the success of PD-1/PD-L1 antibody treatment with or without combination therapies directed against other immune checkpoint modulators depends primarily on the state of the TME. This TME can be strongly immunogenic (hot), with high expression of PD-L1 and cytokines, or non-immunogenic (cold), with almost no PD-L1 expression and no T cell infiltration [371]. Several combination therapies with PD-1/PD-L1 antibodies are being explored to obtain synergistic responses. This combination may include PD-1/PD-L1 antibodies to be used along with other immune checkpoint inhibitors (e.g., CTLA-4 antibodies), neoantigen tumor vaccines, antiviral drugs, anti-microbiome modulators, chemotherapy, and radiation therapy [67]. These newer ICI targets and combination therapies are at an initial phase and showing very promising results. Therefore, further research is needed to assess their safety and efficacy so as to develop better treatment modalities for cancer.

This review has touched upon some of the newer ICI targets but not all of them. There exist other promising targets, such as PVRIG/PVRL2, ICOS, GITR, etc., which are currently being tested in the clinic but have not been expanded upon in this review due to limitations of space [372]. Other, newer targets and pathways are being discovered in various pre-clinical cancer models that hold immense cancer therapy potential. Such examples include, but are not limited to, the KIR3DL3-HHLA2 axis, the KIR2DL5/PVR pathway, GPR56, and HVEM/BTLA [373,374,375,376].

In addition to the newly developed targets mentioned above, other strategies in immunotherapy are gaining prominence and showing promising results in pre-clinical and clinical studies. These studies include the important role of microRNAs, mRNAs, and CRISPR-Cas9 technology, as immunotherapeutic strategies.

MicroRNAs (miRNAs) are small non-coding RNAs that regulate gene expression at the posttranscriptional level, usually by binding to the 3′-UTR of mRNAs [377]. Apart from their multifunctional roles in various physiological and pathological processes of the immune system, miRNAs have been demonstrated to strongly influence immune checkpoint (IC) genes, which in turn have made them an attractive candidate to develop as an immunotherapeutic agent in combination with ICIs [378]. Besides conventional markers to predict the response to ICIs, such as PD-L1 expression and tumor mutational burden, pretreatment serum miRNAs have also found use as a non-invasive diagnostic marker to select patients most likely to benefit from immune checkpoint blockade [379,380]. One study demonstrated that circulating fibroblast growth factor receptor 1 (circFGFR1), functioning as a miRNA sponge of miR-381-3p, induced therapeutic resistance to PD-1 blockade [381]. Nakahara et al. reported that significantly high levels of miR-16-5p, miR-17-5p, and miR-20a-5p marked the responders among melanoma patients receiving anti-PD-1 therapy [382]. Therapy with miRNA can either lead to the restoration of miRNA function (miRNA mimics) or the downregulation of miRNA function (miRNA repression). Delivery systems for miRNAs can be local (i.e., injected directly to the tumor site or delivered topically to the skin) as well as systemic (i.e., via viral vectors, exosomes, or nanoparticles) [383]. Both modes of delivery have faced many challenges since their inception, and, in order to achieve the desired therapeutic effect of miRNA, several obstacles, such as avoiding degradation in the bloodstream, tumor tissue penetration, adequate cellular uptake, and the prevention of off-target effects, must be tackled effectively [384]. Some phase I clinical trials have tested the therapeutic delivery of miRNAs to cancer patients, with modest results. For example, Beg et al. reported the therapeutic use of a liposomal miR-34a mimic (MRX34) for advanced solid tumors refractory (NCT01829971) to all standard treatments, resulting in partial remission, but it lead to dose-limiting renal, pulmonary, and gastrointestinal side effects [385]. In another phase 1 trial, miR-16 was used via delivery in “minicells” targeted to EGFR (called TargomiRs) in patients with advanced mesothelioma (NCT02369198) resistant to standard therapy, but the success of partial remission was overshadowed by side effects of severe cardiac toxicity, fever, and chills [386] Another trial with a miRNA-based approach used cobomarsen, an inhibitor of miR-155, in patients with mycosis fungoides (NCT02580552 clinicaltrials.gov). Cobomarsen has shown potent activity against mycosis fungoides and diffuse large B cell lymphoma and is under evaluation [387,388]. At present, the clinical trial data are somewhat discouraging; however, these trials have brought about relevant translational observations. However, with growing pre-clinical evidence interconnecting immune checkpoint blockade and miRNA therapy, the future holds a great deal of promise.

mRNA therapy is a unique anticancer strategy based on in vitro transcription (IVT), which has shown great promise for the treatment of malignant tumors. mRNA vaccines have emerged as a novel class of cancer vaccines, capable of encoding and expressing tumor-associated antigens (TAAs) or tumor-specific antigens (TSAs) and their associated cytokines [389]. Developing effective delivery systems poses a major challenge to mRNA therapy and, as such, the FDA has approved mRNA vaccines using lipid nanoparticles (LNP) delivery platforms, which are effective for both cell-mediated and humoral immunity [390,391]. mRNA-based vaccines based on IVT are gradually being produced for several tumor treatments. As of now, mRNA cancer vaccines have been classified as encoding TSAs, TAAs, antibodies, and cytokines depending on the type of final product. The majority of clinical trials have applied mRNA cancer vaccines to treat aggressive, refractory, and metastatic tumors. Interim analysis derived from a phase I trial (NCT02410733) demonstrated that BNT111 (the lead product candidate from BioNTech’s fully owned FixVac platform) is a potent immunotherapy in melanoma patients already treated with immune checkpoint inhibitors [392]. mRNA vaccines that encode a large number of mutated antigens show great promise for treating mutation-induced malignancies. Phase I trial data showed that mRNA-4157 (a Moderna vaccine) monotherapy or in combination with the PD-1 inhibitor Keytruda (NCT03313778) was effective by triggering a neoantigen-specific T cell response and had minimal side effects at all doses tested. Promising phase I trial results led to a phase II clinical trial involving the personalized cancer vaccine mRNA-4157 (NCT03897881) for the treatment of cutaneous melanoma [393]. Personalized mRNA vaccines are now the target of future research for developing precision tumor treatments.

The CRISPR-Cas9 system inactivates genes at the DNA level, which enables the phenotypic study of a loss of gene function to be elicited. Additionally, this technology provides the functional interrogation of non-transcribed, inaccessible elements employing RNA interference. Loss-of-function genetic screens have increasingly been used to study the functional consequences of gene deletion in tumor cells [394,395,396]. This approach includes the pooled genetic screens using CRISPR-Cas9-medicated genome editing, which can potentially evaluate the role of tumor cell growth, viability, or drug resistance [397]. In 2017, Manguso et al. demonstrated that CRISPR-Cas9 genome editing in transplantable tumors in mice treated with immunotherapy with PD-1 checkpoint blockade improved the immunotherapy targets. By testing 2368 genes expressed by melanoma cells, they identified that tumors were sensitized to immunotherapy by the deletion of genes involved in several diverse pathways, including NF-κB signaling, antigen presentation, and the unfolded protein response. Additionally, deletion of the protein tyrosine phosphatase PTPN2 in tumor cells increased the efficacy of the immunotherapy by enhancing the interferon-γ-mediated effects on antigen presentation and growth suppression [396]. CRISPR-Cas9 genome knock-in was also efficiently introduced by Eyquem et al. in 2017, with the recent development of a CD19-specific chimeric antigen receptor (CAR) to the TRAC locus (T cell receptor α constant), which results in uniform CAR expression in human peripheral blood T cells. Additionally, it also enhances T cell potency with the edited cells, which immensely outperformed the conventionally generated CAR T cells in a mouse model of acute lymphoblastic leukemia [398]. Stadtmauer et al. demonstrated, in a phase I first-in-human pilot study (clinical trial; NCT03399448), the initial safety and feasibility of multiplex CRISPR-Cas9 T cell human genome engineering in patients with advanced, refractory cancer [399]. It was found that HLA-E was more frequently expressed than PD-L1 in several types of cancer, including Merkel cell carcinoma, and, additionally, the in vivo CRISPR screening knock-out of Qa1b led to the increased efficacy of immunotherapy by PD-1 blockade. These results highlight the need for combination therapy strategies using a combination of mAbs blocking the PD-x and NKG2A/HLA-E inhibitory pathways [17,153,396,400]. Xiao Zhang et al., in 2021, also provided vital information in the field of immunotherapy, namely that the anti-PD-1 response was significantly enhanced in tumors lacking both TAP1 and Qa-1b by knocking out these genes using CRISPR-Cas9 gene editing technology in a B16-F10 melanoma cell line and mice model [401]. The ongoing clinical trials (NCT04426669 (phase 1/2), NCT04035434 (phase 1/2)) on immunotherapeutic agents that are associated with CRISPR-Cas9-mediated cancer immunotherapy include tumor-infiltrating lymphocyte cells for gastrointestinal epithelial cancer, colorectal cancer, pancreatic cancer, gallbladder cancer, and esophageal cancer. NCT03398967 and NCT03166878 (both at phase 1/2), designed to target CD19, CD20, and CD22 on B cells, are associated with B cell leukemia and B cell lymphoma immunotherapy [402]. Additionally, clinical trials with CRISPR-Cas9-mediated PD-1-knock-out autologous T cells are also underway for prostate cancer (NCT02867345), bladder cancer (NCT02863913), and renal cell carcinoma (NCT02867332) [403].

A deeper and better understanding of the novel inhibitor pathways being currently studied is warranted. Moreover, further exploration of the miRNA, mRNA, and CRISPR-Cas9-mediated cancer immunotherapeutic approaches is also needed. The discovery of more targets and other such cutting-edge approaches will help to improve the applications of cancer immunotherapy and will aid in designing future clinical trials, expanding the spectrum of cancer patients benefitting from cancer immunotherapy.

## Figures and Tables

**Table 1:** Important clinical trials pertaining to the immune checkpoint targets mentioned in this review.

Molecule/Target	Drug	Mechanism	Cancer Type	Trial Name/Phase	Estimated/Actual Completion Date
**PD-1**	Retifanlimab + INCAGN02385 + INCAGN02390	Anti-PD-1 + Anti-LAG-3 + Anti-TIM-3	Head and Neck Cancer	(NCT05287113)/Phase 2	15 September 2024
	Pembrolizumab	Anti-PD-1	Refractory melanomas, Non small cell lung carcinoma (NSCLC), Urothelial Carcinoma, Metastatic Head and Neck squamous cell carcinoma (HNSCC) etc.	KEYNOTE Trials: (NCT01295827) Phase 3	11 December 2018
	Nivolumab	Anti-PD-1	Metastatic Melanoma NSCLC, Urothelial carcinoma, Colorectal carcinoma	Checkmate studies (NCT02388906) (NCT01668784) Phase2/Phase3	30 January 2026 19 July 2021
	Cemiplimab	Anti-PD-1	Metastatic pancreatic cancer	(NCT04177810)	1 August 2023
			Malignant glioma	(NCT03690869)	20 November 2024
			Hepatocellular carcinoma	(NCT03916627)	3 September 2029
			NSCLC	(NCT03580694)	4 December 2019
			Renal cancer	(NCT02394083)	5 November 2023
			Lymphoma	(NCT02651662)	19 August 2026
			Multiple myeloma	(NCT03194867)	31 March 2023
			Prostate cancer	(NCT03951831)	December 2022
			Ovarian cancer	(NCT03564340)	December 2022
			Cervical cancer	(NCT03257267)	9 July 2023
				Phase 2/Phase3	
**PD-L1**	Atezolizumab	Anti-PD-L1	NSCLC	NCT02008227/Phase3	9 January 2019
	Avelumab	Anti-PD-L1/PD-1	Renal cell carcinoma	NCT02684006/ Phase 3	21 May 2024
	Durvalumab	Anti-PD-L1	Urothelial carcinoma NSCLC	NCT01693562 NCT02125461	28 February 2020 30 December 2022
**CTLA-4**	Anti-CTLA-4 Monoclonal Antibody BMS-986218 + Nivolumab	Anti-CTLA-4 + Anti-PD-1	Advanced Lung Carcinoma, Advanced Malignant Solid Neoplasm, Malignant Adrenal Gland Neoplasm, Metastatic Liver Carcinoma, Metastatic Lung Carcinoma, Metastatic Malignant Solid Neoplasm	NCT04785287/Phase 1, Phase 2	27 May 2024
**HLA-E**	TTX-080	Anti-HLA-E	Refractory solid malignancies such as HNSCC, NSCLC, Colorectal cancer and triple negative breast cancer	NCT04485013/Phase 1	1 June 2024
**NKG2A**	Monalizumab + Cetuximab + Anti-PD(L)-1	Anti-NKG2A + Anti-EGFR	Head and Neck Neoplasms	NCT02643550/Phase 1, Phase 2	September 2022
	Durvalumab + Monalizumab	Anti-PD-L1+ Anti-NKG2A	Stage III Non-small Cell Lung Cancer Unresectable	NCT03822351/Phase 2	21 June 2023
**NKG2D**	CM-CS1/CYAD-01	CAR T cell	Acute Myeloid Leukemia, Multiple Myeloma, Myelodysplastic Syndromes	NCT02203825/ Phase 1	March 2018
**A2AR & A2BR**	AZD4635 + Oleclumab + Durvalumab	Anti-A2aR & A2bR + Anti-CD73+ Anti-PD-L1	Prostate Cancer Metastatic Castration-Resistant Prostate Cancer (mCRPC)	NCT04089553/Phase 2	31 December 2023
	Etrumadenant + zimberelimab+ mFOLFOX-6 + bevacizumab + regorafenib + AB680	Anti-A2aR and A2bR+ Anti-PD-1 + Anti-CD73	Metastatic Colorectal Cancer	NCT04660812/Phase 1, Phase 2	18 December 2023
**SIRPα/CD47**	CC-90002	Anti-CD47	Acute myeloid leukemia	NCT02641002/Phase1	18 July 2018
	HX009	Anti-CD47/PD-1 bifunctional antibody	Unresectable locally advanced/ metastatic solid tumors	NCT04886271/Phase 2	10 February 2023
	TTI-621	Anti-SIRPa	R/R Hematologic malignancies and selected solid tumors (PTCL, CTCL)	NCT02663518/Phase 1	31 December 2022
	BI-765063/OSE172	Anti-SIRPa	Advanced solid tumors (NSCLC, TNBC, pancreatic cancer, melanoma, HNSCC, RCC, UC, SCL, gastric cancer, CRC and OC)	NCT03990233/Phase 1	31 December 2022
**TIM-3**	TSR022	Anti-TIM-3	High Grade serous ovarian cancer	NCT04139902/Phase 1	October 2027
**LAG-3**	IERAMILImAB (LAG525)	Anti-LAG-3	Advanced/Metastatic solid tumors	NCT02460224/Phase 1	31 December 2020
	Relatlimab	Anti-LAG-3	Previously untreated metastatic/uresectable melanoma	NCT03470922/Phase 1/2	16 December 2025
	REGN3767/Fianlimab + Cemiplimab	Anti-LAG-3 + Anti-PD-1/PD-L1	Previously Untreated Unresectable Locally Advanced or Metastatic Melanoma	NCT05352672/Phase 3	20 April 2031
**B7-H3**	Enoblituzumabn (MGA271)	Anti-B7-H3	Prostate Cancer	NCT02923180/Phase 2	30 July 2023
	MGD009/Orlotamab	Anti-B7-H3	Mesothelioma, Bladder Cancer, Melanoma,Squamous Cell Carcinoma of the Head and Neck,NSCLCr,Clear Cell Renal Cell Carcinoma Ovarian Cancer,Thyroid Cancer, Breast Cancer,Pancreatic Cancer,Prostate Cancer, Colon Cancer, Soft Tissue Sarcoma	NCT04145622/Phase 1	1 December 2023
	MGC018 +/− MGA012	Anti-B7-H3 +/− Anti-PD-1	Squamous Cell Carcinoma of Head &Neck, Triple Negative Breast Cancer, Melanoma, Advanced Solid Tumor, Adult Metastatic Castrate Resistant Prostate Cancer, NSCLC	NCT03729596/Phase 1, 2	May 2023
**PARPs**	Olaparib (AZD2281)	PARP inhibitor	Patients with ovarian cancer that recurred within 12 months of prior platinum therapy and with confirmed germline BRCA1/BRCA2 mutation	NCT00753545/Phase 2	29 December 2023
	Rucaparib	PARP inhibitor	Advanced gynecologic cancer and triple negative breast cancer	NCT03101280/Phase 1	11 August 2020
**TIGIT**	COM701 in combination with BMS-986207 and nivolumab.	Anti-TIGIT Antibody	Endometrial Neoplasms,Ovarian Cancer,Solid Tumor,Head and Neck Cancer	NCT04570839/Phase1 Phase 2	December 2023
	Ociperlimab (BGB-A1217) + Tislelizumab	Anti-TIGIT Antibody	Locally Advanced and Metastatic Solid Tumors	NCT04047862/Phase 1	October 2024
	Tiragolumab + Atezolizumab	Anti-TIGIT Antibody	Non-small Cell Lung Cancer	NCT03563716/Phase 2	30 September 2023
**VISTA**	JNJ-61610588	Anti-VISTA	Advanced Cancer	NCT02671955/Phase 1	July 2017
	CA-170	Oral PD-L1, PD-L2 and VISTA Checkpoint Antagonist	Advanced Solid Tumors or Lymphomas	NCT02812875/Phase 1	7 May 2020
	CI-8993	Anti-VISTA	Solid Tumor	NCT04475523/Phase 1	1 July 2023
**microRNA**	Cobomarsen	Immunotherapeutic MicroRNA	Cutaneous T-cell Lymphoma Mycosis Fungoides Chronic Lymphocytic Leukemia Diffuse Large B-Cell Lymphoma, ABC Subtype Adult T-Cell Leukemia/Lymphoma	NCT02580552/Phase 1	6 October 2020
	TargomiRs	miR-16 Mimic	Malignant Pleural Mesothelioma Non-Small Cell Lung Cancer	NCT02369198/Phase 1	4 January 2017
**mRNA**	Lipo-MERIT	RNA-lipoplex Cancer Vaccine	Melanoma	NCT02410733/Phase 1	May 2023
	mRNA-4157 + Pembrolizumab	Personalized cancer vaccine	Solid Tumors	NCT03313778/Phase 1	30 June 2025
**CRISPR/CAS9**	Tumor-Infiltrating Lymphocytes (TIL)	Tumor Infiltrating Lymphocytes in Which the Gene Encoding the Intracellular Immune Checkpoint CISH Is Inhibited	Gastrointestinal Epithelial Cancer, Gastrointestinal Neoplasms, Gastrointestinal Cancer, Colo-rectal Cancer, Pancreatic Cancer, Gall Bladder Cancer, Colon Cancer, Esophageal Cancer, Stomach Cancer	NCT04426669/Phase 1	January 2023
	CTX110	CD19-directed chimeric antigen receptor (CAR) T cell immunotherapy	B-cell Malignancy, Non-Hodgkin Lymphoma, B-cell Lymphoma, Adult B Cell ALL	NCT04035434/Phase 1	August 2026

Clinical trials as of 22 January 2023 listed on https://clinicaltrials.gov/. The name (when available) and the ClinicalTrials.gov identifier (NCT number) are reported as on the website.

## Data Availability

Not applicable.

## Abbreviations

A2AR	adenosine A2A receptor
ADCC	antibody-dependent cellular cytotoxicity
AML	acute myeloid leukemia
APC	antigen-presenting cells
ART1	mono-ADP-ribosyl transferase 1
ATC	activated T cells
ATP	adenosine triphosphate
(CAR)-T	chimeric antigen receptor T cell
CD	cluster of differentiation
CRC	colorectal cancer
CREB	cAMP response element-binding protein
CRISPR	clustered regularly interspaced short palindromic repeats
CTL	cytotoxic T lymphocytes
CTLA-4	cytotoxic T-lymphocyte-associated protein 4
DC	dendritic cells
DSB	double-strand break
EMT	epithelial-to-mesenchymal transition
FGL1	fibrinogen-like protein 1
Gal-9	galectin-9
GITR	glucocorticoid-induced tumor necrosis factor receptor-related protein
GPCR	G-protein-coupled receptor
HAVCR2	hepatitis A virus cellular receptor 2
HCC	hepatocellular carcinoma
HDAC	histone deacetylase
HIF	hypoxia-inducible factor
HLA	human leukocyte antigen
HMGB1	High Mobility Group Box 1
HNSCC	head and neck squamous cell carcinoma
IAP	integrin-related protein
ICB	immune checkpoint blockade
ICI	immune checkpoint inhibitor
ICOS	inducible T cell COStimulator
IFN γ	interferon-γ
IG	immunoglobulin
IL-10	interleukin-10
IL-15R	interleukin15 receptor
IL-2	interleukin-2
IL-4	interleukin-4
ITIM	immune receptor tyrosine-based inhibitory motif
ITSM	immune receptor tyrosine-based switch motif
LAG-3	lymphocyte activation gene-3
MAR	mono-ADP-ribosyl
mART	mono-ADP-ribosyl transferase
MDSC	myeloid-derived suppressor cells
MHC	major histocompatibility complex
miRNA	micro-RNA
mRNA	messenger RNA
MMP	matrix metalloproteinases
mAb	Monoclonal antibody
NECA	5′-(N-ethylcarboxamido) adenosine
NHL	non-Hodgkin’s lymphoma
NICD	NAD+-induced cell death
NK	natural killer
NKAE	activated and expanded NK cells
NKG2D/A	natural killer group 2D/A
NKG2DL	NKG2D ligand
NSCLC	non-small-cell lung cancer
ORR	objective response rate
MAR	poly-ADP-ribosyl
PARP	poly-ADP-ribose polymerase
PD-1	programmed cell death protein 1
PD-L1/2	programmed cell death ligand 1/2
PI3K	phosphatidylinositol 3-kinase
PKD2	protein kinase D isoform 2
PRR	pattern recognition receptor
PSGL-1	P-selectin glycoprotein ligand 1
Ptdser	phosphatidylserine
PTEN	phosphatase and TENsin homolog
PTPase	protein tyrosine phosphatases
SCLC	small cell lung cancer
SIRPα	signal regulatory protein α
STING	stimulator of interferon genes
TAA	tumor-associated antigen
TCR	T cell receptor
TIGIT	T cell immunoreceptor with immunoglobulin and ITIM domain
TIL	tumor-infiltrating lymphocyte
TIM-3	T cell immunoglobulin and mucin domain 3
TLR7	toll-like receptor 7
TME	tumor microenvironment
TNBC	triple-negative breast cancer
TNF α	tumor necrosis factor-α
Treg	regulatory T cell
TRIM	T cell interacting molecule
TSA	tumor-specific antigen
VEGF	vascular endothelial growth factor
VISTA	V-domain Ig suppressor of T cell activation
WNT	wingless-related integration site
